# Investigation into the role of layered nanoporous modified clays for adsorption/desorption of rosemary essential oil in the gas phase and the ability of this system as an antioxidant and preservation agent in food applications

**DOI:** 10.1186/s40643-025-00884-7

**Published:** 2025-06-06

**Authors:** Doha Berraaouan, Mohamed Tirinsi, Ramzi A. Mothana, Hanan M. Al-Yousef, Marie-Laure Fauconnier, Samira Salhi, Abdesselam Tahani

**Affiliations:** 1 Physical Chemistry of Natural Substances and Process Research Team, Laboratory of Applied Chemistry and Environment (LCAE-CPSUNAP), Faculty of Sciences, Mohammed First University, 60000 Oujda, Morocco; 2 Laboratoire d’Amélioration des Produits Agricoles, Biotechnologie et Environnement, Faculty of Sciences, Université Mohamed Premier, BV Mohammed VI, BP 717, 60000 Oujda, Morocco; 3 Centre de l’Oriental des Sciences et Technologies de l’Eau et de l’EnvironnementUniversité Mohamed Premier, BV Mohammed VI, BP 717, 60000 Oujda, Morocco; 4 Department of Pharmacognosy, College of Pharmacy, King Saud University, 11451 Riyadh, Saudi Arabia; 5 Laboratory of Chemistry of Natural Molecules, Gembloux Agro-Bio Tech, University of Liege, Liege, Belgium

**Keywords:** Adsorption, Montmorillonite clay, Rosemary essential oil, Physisorption, Modelization, Antioxidant activity

## Abstract

A new green composite material with antioxidant properties and specific-targeting capabilities was developed using Montmorillonite Clay in modified forms for rosemary essential oil gaseous adsorption without harmful chemicals or excessive heat. The modified clays were tested for their adsorption capabilities, with the sodic clay showing the highest affinity for the essential oil. The study also found that the adsorbed essential oil on sodic clay had the highest antioxidant activity compared to the organic and pillared clays. Adsorption isotherms revealed values of 40, 30, and 18 mg g^−1^ for sodic, organic, and pillared clay respectively. To assess the experimental data three models were used: First Order, Second Order, and Intra-particular Diffusion. According to the models’ data, the Second Order model fits the experimental results. These results suggest combining modified clays with essential oils can create materials suitable for controlled release systems in food and pharmaceutical applications.

**Highlights**

- The capacity of gaseous adsorption of REO on sodic, organic, and pillared clays depends essentially on the interaction between the clay’s external surface and the essential oil-active molecules.
- Adsorbed essential oil on clays’ surfaces without any direct contact shows an antioxidant activity against DPPH that can be explored in further research concerning food packaging, drug delivery…etc.
- The desorbed amount of REO is practically less than half of the adsorbed quantity which involves both physisorption and chemisorption interactions.

**Graphical Abstract:**

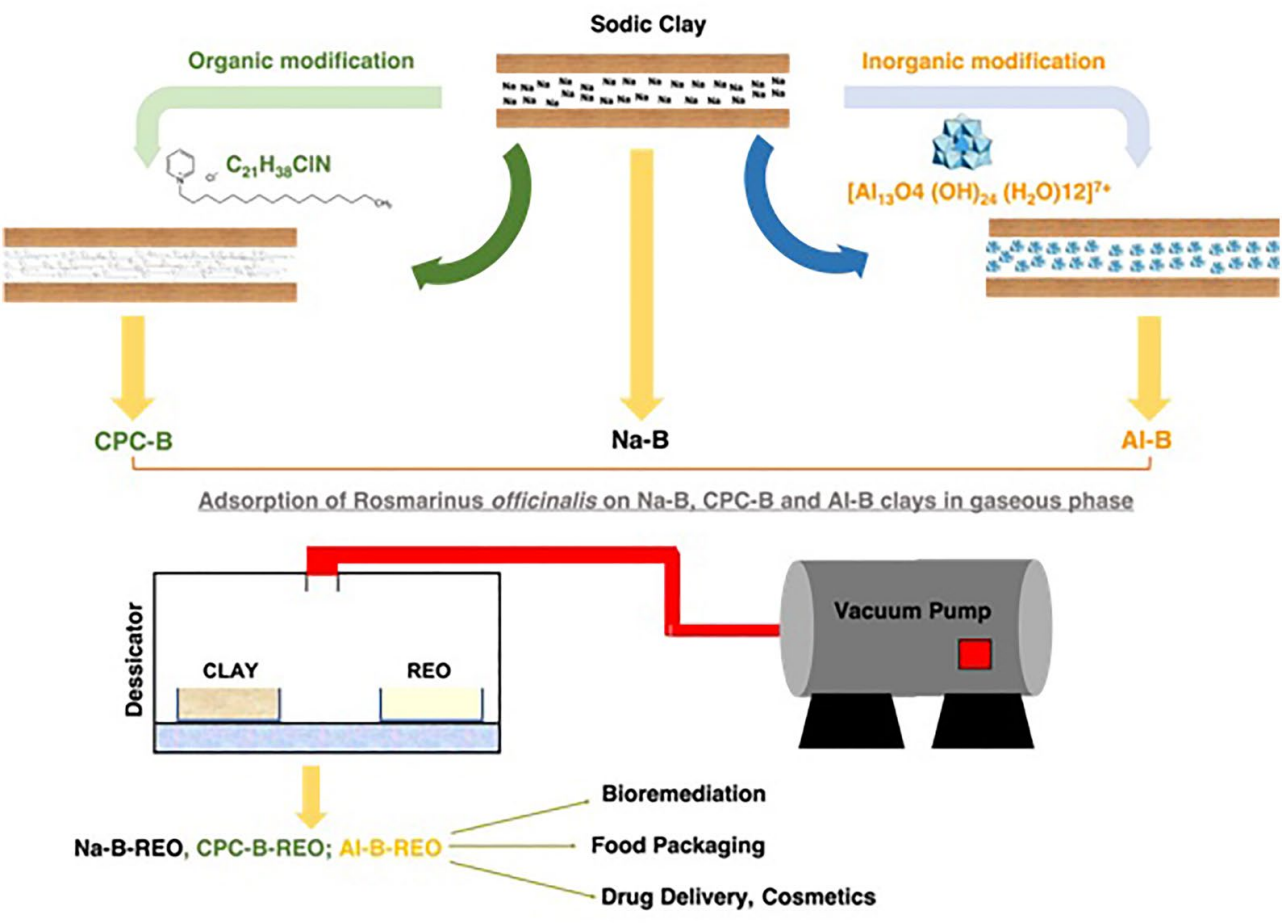

## Introduction

The process of adsorption has extensive applications in various fields, including drug delivery, the food industry, gas storage, catalysts, wastewater treatment, air purification, and separation.

Adsorption is divided into two common types, physical adsorption and chemical adsorption, depending on attractive interactions between the adsorbate molecules and the adsorbent surface ruled by strong or weak intermolecular forces.

Physical adsorption or physisorption is reversible and is caused by weak intermolecular Van der Waals interactions between the adsorbate molecules and the adsorption sites on the adsorbent material. On the other hand, chemical adsorption or chemisorption occurs when the molecules of the adsorbate and the adsorbent surface form a strong, chemical bond that is typically irreversible (Kennedy et al. 2018).

The approach of active molecules adsorption on clays involves reversible attachment of the active molecules into/onto the nano-clay particles by incorporation or adsorption. In other words, the active molecule can fill into the clay matrix and induce changes to the support or can be adsorbed on the surface without any changes to the clay structure depending on the characteristics of the clay (surface area, pore size distribution, charge… etc.) and the active molecules (size, shape, polarity… etc.). The active molecules that are adsorbed, as well as the type of clay utilized, may differ based on the specific objectives of the process. Typically, these active molecules are predominantly present in essential oils in their natural state. It is important to highlight that the conditions facilitating adsorption significantly influence the development of this system; thus, a comprehensive analysis of these conditions necessitates theoretical investigations (Oliveira et al. 2022).

Essential oils (EOs) are fragile, volatile substances (Scalvenzi et al. 2024) that are naturally produced by plants as a by-product of their metabolism. EOs have gained a lot of attention and are being used extensively in the food industry and as biological agents with antiviral, antioxidant, and anti-inflammatory properties thanks to the synergetic effect of their components (Bakkali et al. 2008; Shaaban and H., Farouk, A. 2022). The chemical makeup of these substances consists of a intricate mixture of nonvolatile elements, including fatty acids, hydrocarbons, and carotenoids, which constitute a minor fraction of their overall mass. In contrast, the predominant portion of their weight is comprised of naturally volatile compounds such as terpenoids, alcohols, aldehydes, and esters. Essential oils (EOs) are widely used in aromatherapy, applied topically and sometimes even taken internally (Ali et al. 2015). Reviews on the properties of EOs can be found in the literature (Unalan et al. 2021). Because of their composition, EOs are unstable in the environment and are prone to degradation. They are also volatile at ambient temperature, have low water solubility, and are sensitive to light, humidity, and oxygen. Numerous efforts have been undertaken to maintain them by trapping them in various host matrices (Majeed et al. 2015). Real-world applications often face challenges due to intricate and costly preparation methods, which typically require heat, organic solvents, and matrices that can modify the physical, chemical, and biological properties of essential oils (EO) molecules, thereby restricting their use. To enhance their applicability, it is essential to develop a sustainable adsorption strategy utilizing a suitable host that can safeguard these molecules from oxidation and other forms of degradation. This approach also facilitates controlled release and targeted delivery (Saucedo-Zuñiga et al. 2021).

Research has predominantly focused on the encapsulation of essential oils within organic matrices to regulate their release. However, the direct incorporation of these oils into polymeric materials presents challenges due to their volatile characteristics, which result in a swift migration to the surface and subsequent evaporation, leading to significant loss (Keawchaoon and Yoksan 2011; Maji and Hussain 2008; Parris et al. 2005; Paula et al. 2011). It was proposed that adsorption of EOs onto an inorganic porous material could provide controlled release and protection against polymer processing conditions (Kinninmonth et al. 2013; Elmiz et al. 2019).

In recent discussions, clay nanoparticles have emerged as a promising alternative for the entrapment of essential oils (EOs), primarily due to their accessibility, cost-effectiveness, environmental friendliness, and distinctive physicochemical properties, such as surface charge, substantial specific surface area, and ion exchange capacity, among others. Montmorillonite (Mt), a naturally occurring smectite clay characterized by its large layered structure, has garnered significant attention as an effective carrier for essential oils. The considerable surface area of montmorillonite, resulting from its layered configuration, enhances its efficacy as an adsorbent. The adsorption capabilities of montmorillonite have been extensively studied and documented in scientific literature, showcasing numerous instances of organic material adsorption, including 2,4,6-trichloroanaline (Gianotti et al. 2008) and tetracycline (Parolo et al. 2012). It may be modified using several versatile processes through intercalation of organic compounds, pillarization, acid activation, and thermal treatments. These endow the montmorillonite with new properties and adsorption sites and spaces that enhances the organic component adsorption. This modified montmorillonite may be characterized by a high surface area, organophilicity, hydrophobicity and other singular characteristics. These traits improve its affinity to specific molecules like phenols which is initially limited by several factors (França et al. 2022). These interactions between clays and phenols occur as a result of interfacial reactions, catalysis or complexation with modified clays according to the chemical composition of the phenols and the physical characteristics of clays. Adsorption of EOs onto montmorillonite is a new area of study; a paper by Nguemtchouin et al. (2013) investigated the adsorption of Ocimum gratissimum EO onto Mt and organically modified Mt. A further study examining the adsorption of essential oils onto layered silicates has demonstrated the appropriateness of these materials as carriers for active compounds (Kinninmonth et al. 2013).

In their study, Giannakas et al. (2017) investigated the use of oregano oil, thyme oil, and basil essential oil with a purified and organo-modified montmorillonite for applications involving controlled release while Ghrab et al. (2018a) utilized eucalyptus essential oil in combination with modified beidellite using cationic surfactants for insecticidal purposes. There are multiple ways to load EOs onto unmodified or modified clays. The most common batch approach involves dissolving the EO in an organic solvent before adding it to the clay (Nguemtchouin et al. 2015) or to mix them together and heat them at 120 °C for 24 h (Giannakas et al. 2017; Ghrab et al. 2018b; Essifi et al. 2022). Studies have shown that encapsulated orange and thyme essential oils exhibit excellent adsorption/encapsulation efficiency and biological effectiveness (Giannakas et al. 2017; Essifi et al. 2022).

The majority of the investigations have primarily focused on adsorption studies, whereas desorption studies concerning Mt have not garnered equivalent attention. Nonetheless, there have been instances where controlled desorption has been successfully accomplished through the application of clays (Kim et al. 2005).

The ability to capture active molecules after the adsorption process and to release them is regarded as a vital feature due to its wide-ranging applications as in food packaging where it fulfills two primary functions: the safeguarding of the quality and safety of the product from spoilage while simultaneously prolonging its shelf life. This is achieved by shielding the food from a range of potential damages, which encompass chemical, biological, environmental, and physical hazards encountered during transportation and storage (Arif et al. 2022; Khalid and Arif 2022; Haghighi et al. 2021; Solis and Silveira 2020). An additional significant aspect addressed through the process of bio-preservation is the utilization of environment friendly materials. This approach effectively mitigates the issue of non-biodegradable waste, which is often costly to manage and detrimental to the environment (Hamed et al. 2022).

**Fig. 1:**
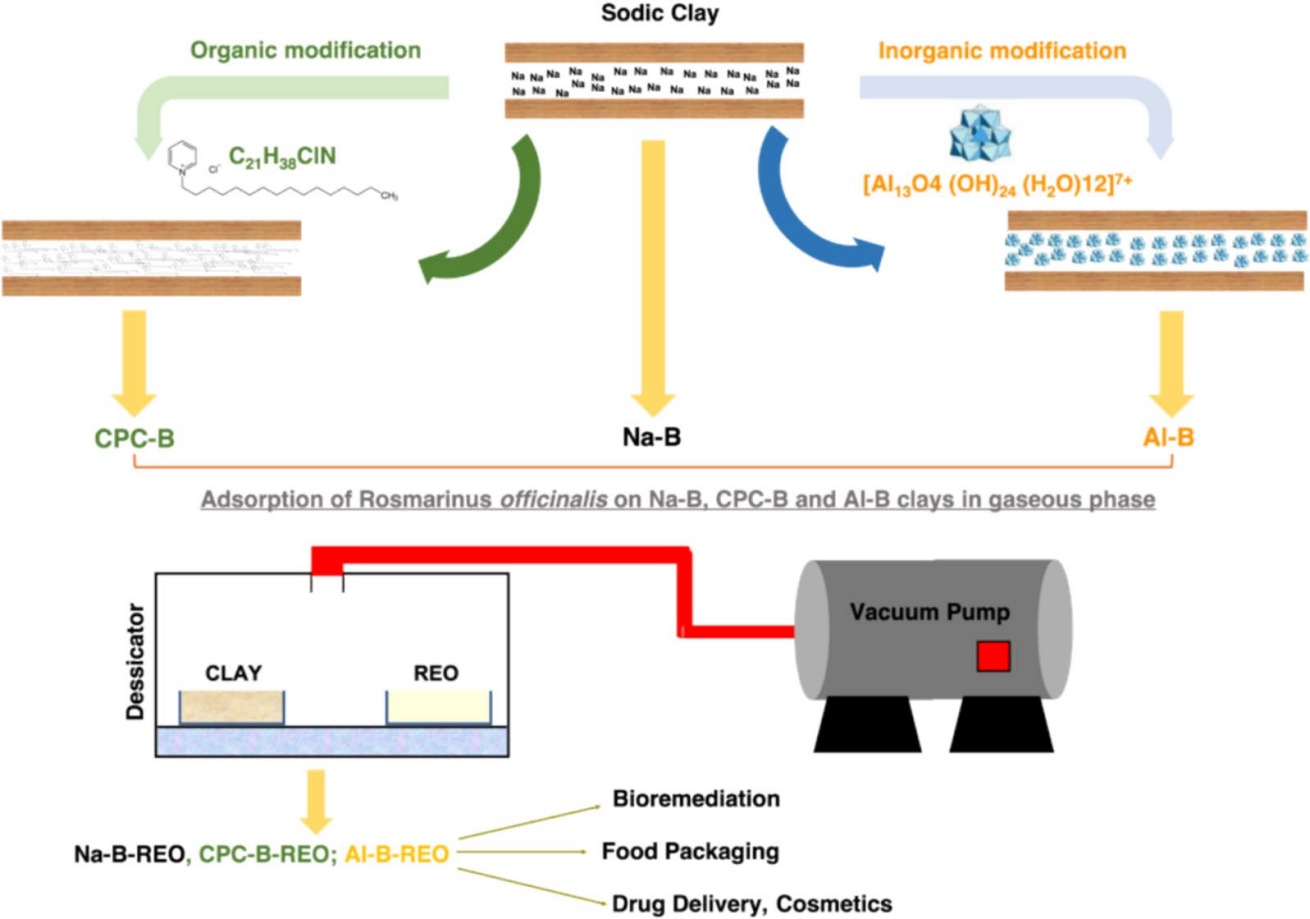
Graphical abstract

The main objective of the present work is to study the adsorption of rosemary essential oil on three different types of clay from the eastern region of Morocco (hydrophilic, hydrophobic and porous pillared clay) and its pathway release as a function of time in gaseous phase using a chemical free method (as shown on Fig. 1) demonstrating that it is possible for a volatile compound to be adsorbed onto a substrate without any direct contact. Crucially, this method facilitates the formation of durable bonds between the two entities, ultimately leading to the development of a novel compound that holds significant potential as an antioxidant agent within the food industry.

X-ray diffraction (XRD), Fourier transform infrared spectroscopy (ATR-FTIR), and thermogravimetric analysis (TGA) were used to characterize the blank clays and the REO-clays. Furthermore, the REO adsorbed clay samples were subjected to an examination using the DPPH free radicals’ assay.

## Materials and methods

### Materials

The raw clay used in this study was collected from Nador (North-East Morocco). The clay was purified, and exchanged using sodium chloride, a cationic surfactant Cetyl Pyridinium Chloride (CPC) and [Al_13_O_4_(OH)_24_(H_2_O)_12_]^7+^ Keggin ion before it was used. CPC used was from HIMEDA, 98%, pure and Aluminium chloride hexahydrate, 99% was from Bernd Kraft. The clay used in this study is a bentonite rich in montmorillonite clay and has been previously described by *El MIZ* (Mohamed et al. 2017).

The essential oil derived from *Rosmarinus officinalis* L. (REO) was sourced from a distillation facility situated in Jerrada, located in the eastern region of Morocco, and was subsequently stored at a temperature of −1 °C until the time of analysis. The essential oil’s characterization has been detailed in earlier research conducted by Berraaouan et al. (2023).

### Preparation of homosodic clay Na-B

Raw bentonite was purified using hydrochloric acid (0.5 M. 37%) and hydrogen peroxide (10% w/v) to remove the organic impurities and those of the crystalline phases. Then, the purified clay was homoionized at a solid:liquid ratio of 1/50 using a 2 M NaCl (99%) solution. After an overnight stirring, the clay underwent a series of rinsing and centrifugation then was dried for no longer than 24 h at 60 °C (Elmiz et al. 2019). Organic and pillared clays were produced using a fraction of the homosodic clay. 

### Preparation of organic clay CPC-B

The synthesis of organic clay followed Srinivasan and Fogler’s method (Srinivasan 1990). Homosodic clay was added to 1% Cetyl-pyridinium chloride solution prepared by dissolving the surfactant in distilled water 1% w/v and left to stir for 12 h at room temperature. Finally, the organic clay was rinsed with distilled water until the foam was diminished then centrifuged and dried at 60 °C. The obtained organic clay was grounded to powder for further use.

### Preparation of pillared clay Al-B

The modification method followed consists on adding a pillaring solution containing [Al_13_O_4_(OH)_24_(H2O)_12_]^7+^ cations at pH = 4.5 into a homosodic clay suspension under stirring for 6 h until complete homogenization. After that, the pillared clay was dried after being centrifuged and filtered and finally, the calcination was carried out at 350 °C for 6 h. The physical characterization of the clays samples is detailed in Table 1.

**Table 1:** The main parameters determined for the different clays

Clay	CEC_EDACu_ (meq/100 g)	BET (m^2^/g)	PoreVolume (cm^3^/g)	ZetaPotential (mV)
Na-B	91.66	94.25	0.28	−24.72
CPC-B	27.89	25.63	0.108	−29.49
Al-B	20.98	107.28	0.104	−18.05

CEC: is the cationic exchange capacity using the copper ethylene diamine complex [Cu (EDA)2]2+ and BET: specific surface of clays

As shown in Table 1, the zeta potential of CPC-B is −29.49 mV, while that of Na-B is −24.72 mV. The phenomenon can be attributed to the surfactant’s positive charges, which increase the overall amount of negative charges present in the clay, thereby inducing electrostatic repulsion. Additionally, the hydrophobic chain of CPC interacts with the negatively charged edges of the clay, creating immobilizing bridges that reduce the flocculation capacity of the clay (Yalçın et al. 2002). On the other hand, the zeta potential drops to -18.05 mV for pillared clay. This results from Al–OH oligomers creating a positive charge on the particle surface (Avena et al. 1990).

### Characterization methods

In order to analyze and identify the raw and modified samples, X-ray diffraction (XRD), Infrared spectroscopy (FT-IR), and Thermal (TGA) analysis are used.

#### Mineralogical analyses by X-ray diffraction

The interlayer space of all Na, CPC and Al-B and Na, CPC and Al-B/EO was estimated from the XRD using a Shimadzu XRD diffractometer D6000 station. The diffractograms were obtained using monochromatic copper K_a1_ radiation (1.54 Å) and a scanning angle of 2 h from 5° to 80°.

#### Attenuated total reflection Fourier transform infrared (ATR-FTIR)

The analysis utilizing attenuated total reflectance-Fourier transform infrared (ATR-FTIR) spectroscopy was performed with a FTIR-8400S SHIMADZU spectrophotometer (Japan) to identify the functional groups present in various clays, rosemary essential oil, and clays combined with rosemary essential oil within the wavelength range of 400 to 4000 cm^−1^. To prepare the samples, a small quantity was mixed with KBr powder, 99% in a 1:9 ratio, then compressed to create a thin pellet, which was subsequently placed in a sample holder to allow infrared radiation to pass through. The spectra obtained for each sample were generated by averaging 32 scans at a resolution of 4 cm^−1^.

#### Evaluation of thermal stability by TGA

The thermogravimetric analysis was performed using a SHIMADZU TA-60WS thermal analyzer (Japan), with each sample having an initial mass of 22 mg. The samples were placed in an alumina holder and subjected to a heating rate of 20 °C/min until reaching 1000 °C under nitrogen atmosphere.

### Adsorption and desorption studies

Under saturated conditions of REO, gas-phase adsorption of the active compounds on the studied clays was carried out using the ethylene glycol adsorption protocol for specific surface area determination (Mulla 1985). Clay samples weighing 1 g each were subjected to heating at 60 °C for an extended period overnight. Following this process, the samples were reweighed and subsequently placed in a vacuum desiccator containing silica gel equipped with a moisture indicator. This setup was employed to eliminate any residual moisture and to ensure that the adsorption sites remained unoccupied by water molecules.

Next, masses of rosemary essential oil, twice the mass of clay, were deposited in the desiccator after removing the silica gel. The samples were maintained in a vacuum environment. The purpose of conducting the experiment under vacuum conditions is to enrich the atmosphere within the desiccator with active compounds, which are known to be volatile. This approach facilitates the investigation of their gaseous diffusion towards the clay while avoiding direct contact.

The adsorbed /desorbed quantity of REO is calculated following the Eq. (1):

1 $${Q}_{ads}/ {Q}_{des}=\frac{{m}_{f}-{m}_{i}}{{m}_{i}}$$

Where ${Q}_{ads}$ is the adsorbed quantity of REO, ${m}_{f} \text{and }{m}_{i}$ are the final and the initial mass after adsorption or desorption. The identification of active compounds within the clays is accomplished through infrared spectroscopic techniques. Following the stabilization of the clay mass, the process of adsorption ceases, indicating that the mineral has reached saturation. To determine the amount of REO desorbed, calculations are made based on the mass difference observed after the clay surface has become saturated. At this juncture, the clays are re-evaluated using infrared spectroscopy.

Three different kinetic models, namely the pseudo-first order (PFO), pseudo-second order (PSO) and intraparticle diffusion (ID), have been utilized to elucidate the adsorption kinetics. The rate constants for the adsorption of REO onto different clays were determined using the expressions of these models. The linear form of the pseudo-first order equation, as represented by Eq. (2), was employed to characterize the adsorption resulting from the ion exchange process (Lagergren 1898):

2 $$\text{log}\left(qe-qt\right)=\text{log}qe-\left(\frac{{k}_{1}}{2.303}\right)t$$

On the other hand, the linear form of the pseudo-second order model, as indicated by Eq. (3), is based on the assumption that the rate-limiting step is the number of active sites available for adsorbent occupation (Ho et al. 1996):

3 $$\frac{t}{{q}_{t}}= \frac{1}{{k}_{2} {q}_{e}^{2}}+ \frac{1}{{q}_{e}} t$$

Finally, the intraparticle diffusion model which describes the diffusion /transport of the REO to the clay is expressed following the Eq. (4) (Weber and Morris 1963):

4 $${q}_{t}= {K}_{dif}^{1/2}+C$$

where qe and qt the adsorbed amount of REO at equilibrium (e) or time (t); K_1_, K_2_ and K_dif_, the adsorption rate constant for PFO, PSO and ID, respectively and C is the intercept.

### Determination of antioxidant activity

The antioxidant activity of clay adsorbed with rosemary essential oil was assessed using 1.1-diphenyl-2-picrylhydrazyl (DPPH 95%), following the procedure outlined by Ling et al. (2022). In summary, a 0.1 mM ethanolic solution (99% HPLC Grade) of 1.1-diphenyl-2-picrylhydrazyl (DPPH) was prepared from which 2.5 mL was forcefully added to a mass of 500 mg of each clay followed by a 30-min dark incubation period at room temperature. The absorbance, determined by a Rayleigh UV–VISIBLE 1800 spectrophotometer (Model Jasco 560, Pekin, China) at 517 nm was used to calculate the IC_50_ of each sample.

## Results and discussion

### Mineralogical analyses by X-ray diffraction

**Fig. 2:**
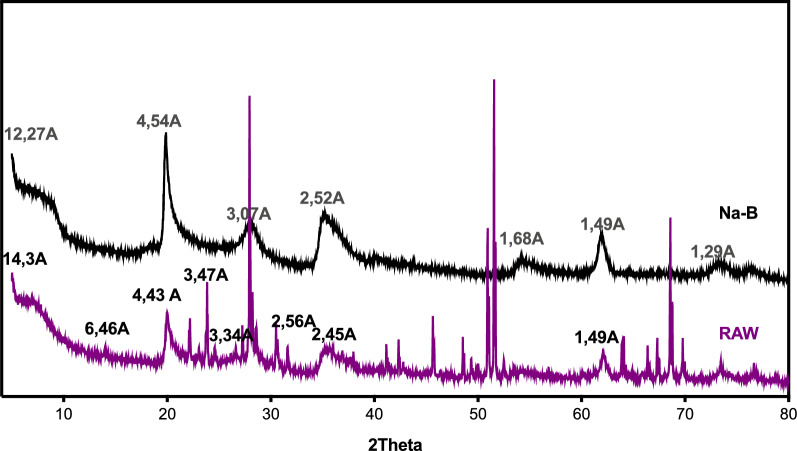
Diffractogram of Na-B and raw clay

**Fig. 3:**
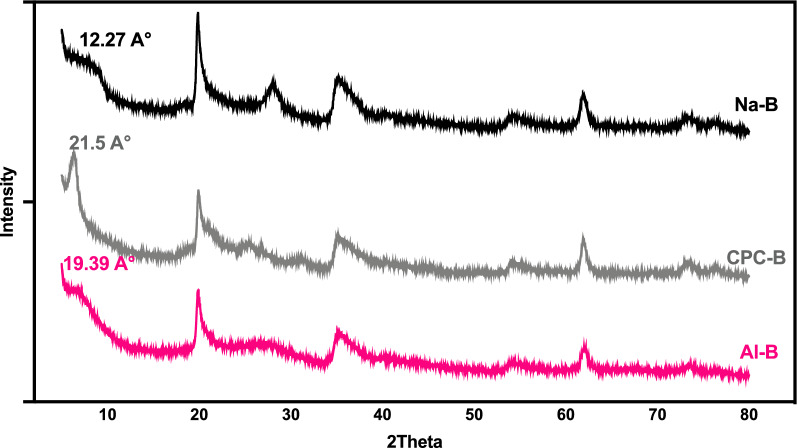
Diffractograms of Na-B, CPC-B and Al-B

Diffractograms of the clays studied are shown in Figs. 2 and 3. The characterization of raw and sodic (Na-B) bentonite using X-ray diffraction (XRD) indicates that the material belongs the smectite family, with reflection (001) concentrated at 14.27 Å. This suggests that bentonite in its raw and natural form is calcic. The line at d = 1.49 Å is present in both clays, indicating that the majority of its composition is made of montmorillonite. At d = 3.07 Å, crystalline phases manifest Quartz (Q) impurities.

The modification process that aims to convert raw bentonite into purified and sodium-exchanged bentonite is verified by analysis of the sodium bentonite diffractogram. The successful exchange of calcium and sodium ions is indicated by the shift of the (001) line from 14.27 to 12.27 Å. The lines that represent the crystalline phases of impurities, namely quartz, are situated at d = 3.34 Å was minimized, whereas certain lines that relate to montmorillonite are intensified at d = 3.07 Å.

Compared to Na-B, which basal spacing is of 1.27 nm (2θ = 6.94°), the basal reflection of organophilic clay (CPC-B) is displaced to a lower angle (2θ = 4.18°), and the basal spacing has increased from 12.27 to 21.5A°. This indicates that the surfactant was successfully intercalated into the sodium bentonite. Because of the length and density of the alkyl chain and the surface charges of the clay, the carbon chain of the alkylammonium group can form either monolayers or bilayers in parallel with the supercoat layer of the clay (Elmiz et al. 2019).

The DRX analyses of the pillared clay show a signal variation at 12.27 Å, which corresponds to the characteristic smectite signal for Na-B at a value close to 19.39 Å, representing an increase in the basal spacing (the inter-foliar space) of the pillared clay. This indicates that the chemical change from sodic clay to pillared clay has taken place. The basal spacing, which expands to around 19.39 Å, is equal to the thickness of a clay layer (9.69 Å) plus the height of an Al_13_ cation (9.7 Å).

### Attenuated total reflection fourier transform infrared (ATR-FTIR)

**Fig. 4:**
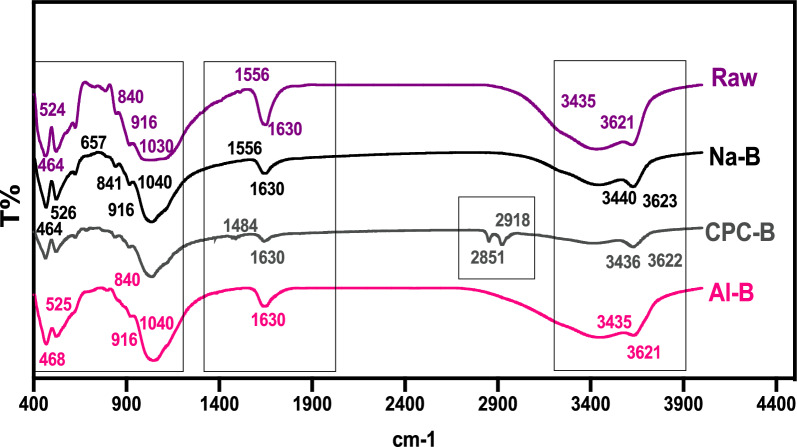
FT-IR spectra of RAW, Na-B, CPC-B and Al-B clays

The results of infrared analysis of the various clay samples are shown in Fig. 4. The examination of the spectra reveals absorption bands as follows:

For raw (Raw) and sodic (Na-B) clays, two absorption bands are observed in the spectrum between 1556 and 1630 cm^−1^ and between 3440 and 3623 cm^−1^. The first is linked to the valence vibrations of the OH grouping of adsorbed and constituted water. The second represents the characteristic band of montmorillonite and corresponds to the vibratory movements of OH elongation in the octahedral layer. The band between 916 and 1030 cm^−1^ corresponds to the valence vibrations of the Si–O bond. In sodic clay, it is centered at around 1040 cm^−1^. The bands between 464 and 524 cm^−1^ are assigned to the deformation vibrations of the Si–O–Mg, Si–O–Fe and Si–O–Al bonds*.*

In the case of the infrared spectrum of organophilic clay (CPC-B), the asymmetrical and asymmetrical C-H stretching movements that appear at 1484, 2852 and 2918 cm^−1^ are attributed to the intercalation of the CPC surfactant in the clay. In addition, we note the disappearance of a bending vibration band signal concerning H–O-H in the CPC-B spectrum, which is present in the raw, sodic and pillared clays at 3435, 3440 and 1630 cm^−1^ respectively. In the case of the pillared clay (Al-B), the bands corresponding to the presence of hydroxyl groups and water molecules in the clay interlayers appear at 3435 cm^−1^.

It can be seen that at 3621 cm^−1^, the intensity of the OH elongation vibration band is a function of the nature of the interlayer cations. For Al-B, the intensity is lower than the intensity of Na-B.

At low frequencies, the infrared spectra of Na-B and Al-B are quite similar. The latter shows a low-intensity band between 468 and 525 cm^−1^ which is due to the folding of Si–O bonds and the elongation movements of Al-O caused by the increased aluminum content of the substrate. The Al-B absorption band at 1630 cm^−1^ corresponds to an increase in water content due to the intercalation of Al–OH cations in the inter-foliar space of the clay.

### Evaluation of thermal stability by TGA

**Fig. 5:**
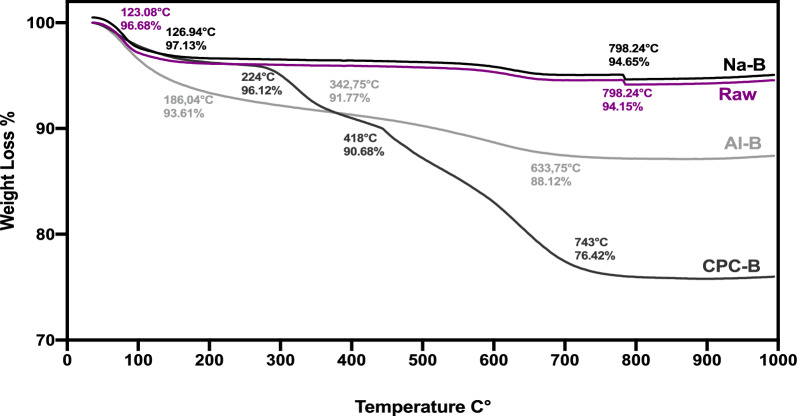
Thermogravimetric analysis of Raw, Na-B, CPC-B and Al-B

The thermograms of the clays studied are shown in Fig. 5. The analysis of the TGA thermal profile for the clays under investigation indicates that the curve for Raw clay exhibits a thermal event at 123 °C, attributed to the release of zeolitic and hygroscopic water. Additionally, at 798 °C, the loss of structural water is observed as a low-intensity endothermic event.

In the low-temperature range, an intense phenomenon occurs at 126.94 °C for Na-B corresponding to the departure of hygroscopic and zeolitic waters from the bentonites. At 798 °C, another less intense phenomenon occurs. It is linked to the departure of structural water (OH groups bound to the edges of clay sheets are eliminated).

Thermogravimetric analyses were conducted on organophilic clay to elucidate the role of CPC in enhancing the hydrophobic properties of the modified clay. The weight loss observed between 224 and 418 °C is attributed to the decomposition of CPC that is adhered to the external surface of the clay. A significant weight reduction occurs in the temperature range of 418 to 743 °C, which is associated with the breakdown of inter-foliar CPC.

In the case of pillared clay, a notable event is detected at 137 °C, indicating the loss of absorbed water. The mass loss in this instance is less than that observed in raw clay, which can be explained by the presence of hydrated cation exchanges within the inter-foliar spaces. Dehydroxylation takes place between 342 and 633 °C, resulting in a stabilization of weight loss, a phase that is indicative of the stability of the pillars. The thermal stability of Al-B pillared clay is corroborated by the findings from the thermogravimetric analysis.

### Adsorption and desorption kinetics

**Fig. 6:**
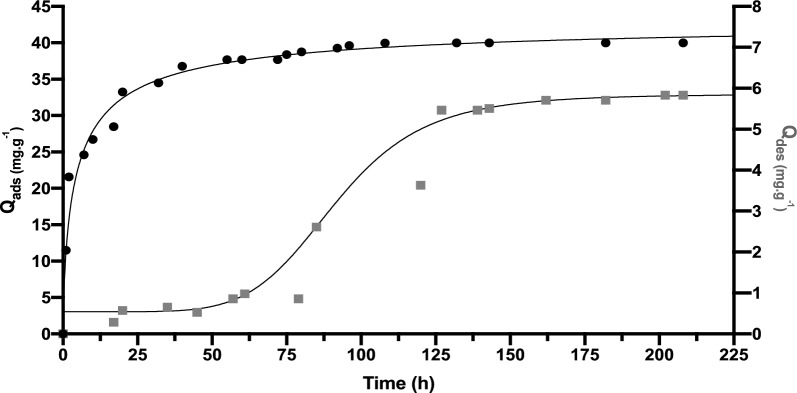
Adsorption (*black circle*) and Desorption (*black square*) kinetics for rosemary essential oil on Na-B in the gas phase

**Fig. 7:**
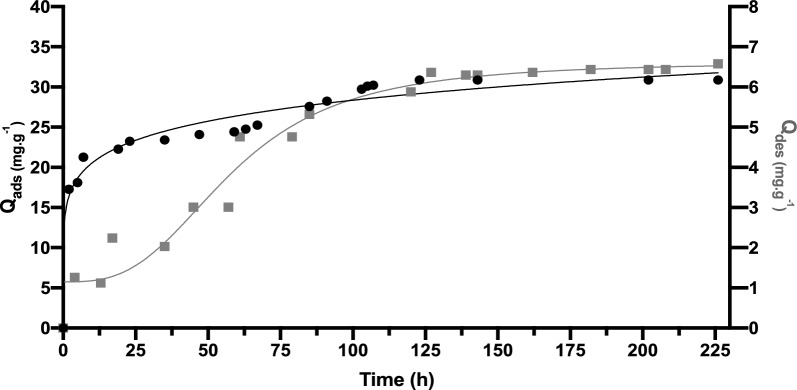
Adsorption (*black circle*) and Desorption (*black square*) kinetics for rosemary essential oil on CPC-B in the gas phase

**Fig. 8:**
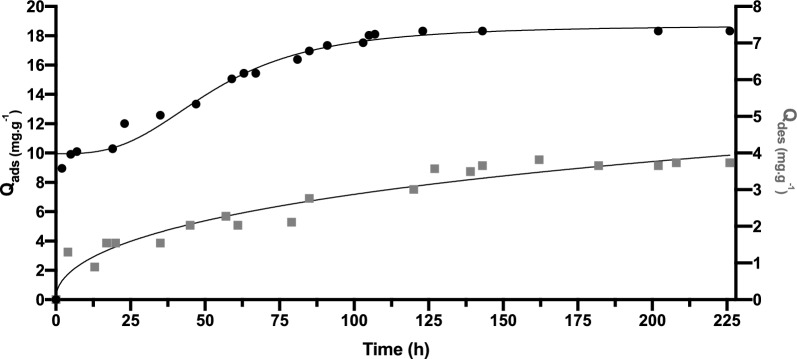
Adsorption (*black circle*) and Desorption (*black square*) kinetics for rosemary essential oil on Al-B in the gas phase

Adsorption and desorption kinetics of rosemary essential oil on the different clays, Na-B, CPC-B and Al-B were studied under vacuum at a system temperature of 22 ±°1 °C. The results are shown in Figs. 6, 7 and 8 for Na-B, CPC-B and Al-B, respectively.

The adsorption–desorption kinetics of essential oil of rosemary on clay surfaces involves several steps, starting with the *Initial Contact*, when the rosemary essential oil comes into contact with the clay surface, it begins interacting with the surface through various forces such as van der Waals forces, hydrogen bonding and electrostatic interactions. Next, the *Adsorption mechanism* by which the molecules of the REO adhere to the clay’s surface depends on the temperature, concentration of the essential oil, pressure, and the characteristics of the clay and oil molecules.

As adsorption proceeds, the system eventually reaches an *Equilibrium*, at which point steady conditions maintain the same quantity of molecules of rosemary essential oil adsorbed on the clay surface. Finally, the *Desorption* is the term used to describe the release of adsorbed molecules from the surface of clay, allowing them to return to their initial environment.

Similar to adsorption, desorption kinetics are influenced by factors like temperature, pressure, and the affinity of the molecules to the clay surface.

The high sodium content of sodic clays plays a significant role in the adsorption process of rosemary essential oil, leading to unique properties. The findings presented in Fig. 6 demonstrate the variation in the quantity of adsorbed REO on sodic clay over time. The examination of the isotherm reveals that the rate of rare earth oxide (REO) adsorption on sodic clay exhibits a rapid increase during the initial hours of interaction, subsequently leveling off as it approaches saturation. The rapid kinetics of adsorption noted at the beginning of the investigation reached 34 mg g^−1^, attributable to the substantial number of active sites available on the sodic clay surface at the onset of adsorption. Subsequently, the remaining vacant sites become increasingly difficult to access due to repulsive interactions between the free REO and the REO already adsorbed on the sodic clay. Equilibrium in the adsorbed quantity is achieved after 37 h, with a final value of 40 mg g^−1^ reached after a contact time of over 200 h between the clay and the REO.

In contrast, the desorption kinetics display a different trend from that of adsorption. There was a consistent release of less than 1 mg/g over a 60-h period, followed by a sudden increase in release from 75 to 125 h, before stabilizing at 5.8 mg.g^−1^. These results indicate the strong affinity of clay for REO molecules and its ability to retain them.

Organic clays possess an increased attraction to organic compounds as a result of their hydrophobic nature following the introduction of cetyl pyridinium chloride. This alteration in chemical composition diminishes the presence of water molecules and facilitates the binding of non-polar and volatile molecules present in essential oils to the surface of the clays. The adsorption process, illustrated in Fig. 7, demonstrates a rapid attainment of equilibrium within 25 h, with subsequent stabilization observed throughout the duration of the study on CPC-B. The quantity of REO adsorbed reached 30.8 mg g^−1^.

It should be expected that organic modifications improve the affinity of clay minerals for rosemary essential oil, yet it’s the non-organic clay with great hydrophilicity that would make the most suitable adsorbent materials for REO, showing the highest adsorbed amount as reported by Kinninmonth (Kinninmonth et al. 2013).

Research into the adsorption of essential oils onto pillared clays is a rich area with potential applications across various industries. To improve the surface area and adsorption potential of natural clays like montmorillonite or bentonite, metal ions or molecules are intercalated within their layers, resulting in the formation of novel modified materials as pillared clays. The adsorption of essential oils onto pillared clays can take place through several mechanisms: Physical adsorption involves weak van der Waals forces binding the essential oil molecules to the surface of the clay particles. The extensive surface area and porous nature of pillared clays provide numerous sites for physical adsorption to occur. Chemical adsorption, on the other hand, occurs when the metal ions or functional groups on the surface of pillared clays chemically interact with the essential oil molecules. This interaction can lead to stronger adsorption through chemical bonding or ion exchange.

Partitioning involves the separation of essential oil components into the interlayer gaps of pillared clays and the aqueous phase. The partitioning behavior is influenced by the characteristics of the clay, as well as the polarity and molecular size of the oil components. Lastly, pore filling occurs when certain components of essential oils enter the pores and interlayer spaces of pillared clays, effectively filling them and facilitating adsorption. The REO composition consisted of hydrocarbon-based molecules, with a CH_3_ group typically measuring 0.4 nm in width (Kadar et al. 2006). Therefore, in order to accommodate the REO molecules, the interlayer space of the substrate would need to be at least 0.4 nm. Considering the usual thickness of a layered aluminosilicate platelet is 0.94 nm (MacEwan 1948). This leads to a consequent reduction in the penetration of REO molecules into the galleries of CPC-B and Al-B, leading to a significant portion of the surface area being inaccessible for adsorption. On the other hand, the enhanced adsorption observed with sodic clay Na-B may be attributed to the presence of Brønsted and Lewis acidic sites on its surface. These sites could facilitate hydrogen bonding between oxygenated species and the clay surface, a phenomenon commonly observed in various REO molecules (Kinninmonth et al. 2013).

Contrary to gaseous phase, silicates have been observed to expand when in a liquid state (Moavenian and Yasrobi 2008). The expansion that occurs during the adsorption process has the potential to enlarge the interlayer space enough to accommodate the EO molecules.

A prior study has indicated that the incorporation of bentonite clay as a nanofiller within biodegradable polymer matrices can significantly enhance the biodegradability of food packaging materials (Maurizzi et al. 2022; Punia Bangar et al. 2023). In a separate investigation, Baniasadi et al. (2025) developed antibacterial and sustainable food packaging films by utilizing carboxymethylcellulose and chitin nanofibrils derived from fungi, reinforced with clay to improve mechanical strength, moisture resistance, and gas barrier properties. To further improve the efficacy of food packaging systems in both environmental protection and food preservation without the use of harmful preservatives, research has shown that free essential oil (EO) molecules can influence the organoleptic properties of food and diminish their antimicrobial and antioxidant effects due to the rapid release of volatile compounds. In contrast, encapsulating these EOs facilitates a controlled release in response to both internal and external stimuli (Maurizzi et al. 2022).

**Fig. 9:**
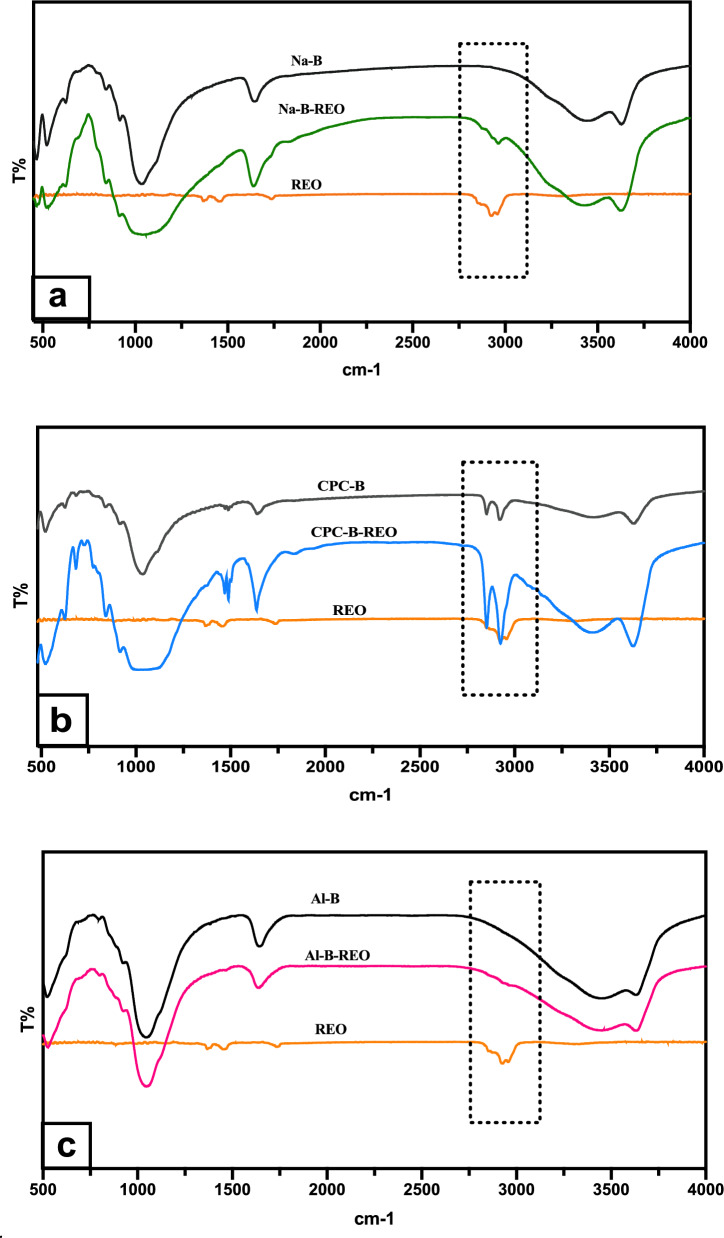
FT-IR spectra of **a** REO, Na-B and Na-B-REO; **b** REO, CPC-B and CPC-B-REO and **c** REO, Al-B and Al-B-REO clay sample*s*

The IR spectra of clays, rosemary essential oil (REO) and clays loaded with REO are shown in Fig. 9. A large number of adsorption bands appear on the spectrum, the most important of which are as follows: The band between 3430 and 3500 cm^−1^ is attributed to the elongation vibration of the O–H phenolic hydroxyl group. The intense band at 2900–3000 cm^−1^ corresponds to the asymmetrical and symmetrical elongation of CH_2_ and CH_3_. Peaks around 1500–1700 cm^−1^ correspond to the C=O and C–C vibrations of the aromatic ring. The bands at 1350–1380 and 1050–1270 cm^−1^ correspond to the deformation of the CH_3_ group of CH_3_(CO) and to the symmetrical and asymmetrical vibrations of C–O–C, respectively. The bands obtained in the 650–1070 cm^−1^ region can be assigned to the out-of-plane deformation of the C-H belonging to the aromatic ring.

Infrared spectra of clays after adsorption of rosemary essential oil show a shift of the 3400–3500 cm^−1^ peak, attributed to OH phenolic vibrations, towards 3000 cm^−1^.

Specifically, the spectra of the sodic clay loaded with REO (Fig. 9a), the fingerprint characteristic of rosemary essential oil (2600–3200 cm^−1^) were observed in the spectra of Na-B-REO clay but were absent in the Na-B clay. Moreover, the peak at 1630 cm-1, associated with the valence vibrations of the OH group of adsorbed and structured water, exhibited increased intensity in Na-B-REO as compared to Na-B clay, indicating potential interactions of inclusion between the clay and rosemary essential oil. The peak at 1630 cm^−1^ which is linked to the valence vibrations of the OH grouping of adsorbed and constituted water was intensified in Na-B-REO compared to the Na-B clay suggesting interactions of incorporation between the clay and rosemary essential oil.

It is necessary to mention that DRX analysis has been done to the clay after adsorption and showed that the Clay-REO pattern is similar to that reported for the different clays before the adsorption in such a way that there are hardly any changes in the profiles of the diffractograms. The absence of changes in the d001 reflection suggests that rosemary essential oil was not intercalated in the interlayer space, which should cause an increase in the basal spacing of the sodic and modified montmorillonites. Thus, this result shows that REO must be adsorbed on the external surface of the studied clays (Kinninmonth et al. 2013).

Modelling of the adsorption kinetics of the three active compounds on clays in gas-medium is summarized in Table 2.

**Table 2:** Modelisation parameters of adsorbed REO on Na-B, CPC-B and Al-B

Pseudo first order							
Clay	R^2^		Value	Std Err	K_1_	q_ex_	q_the_
Na-B	0.8691	Slope	−0.01924	0.001996	−0.01924	40	4.01
		Intercept	1.391	0.1188			
CPC-B	0.8371	Slope	−0.01018	0.001246	−0.01018	30.89	3.35
		Intercept	1.209	0.08199			
Al-B	0.8799	Slope	−0.01286	0.001270	−0.01286	18.33	3.10
		Intercept	1.134	0.08489			

Pseudo second order							
Clay	R^2^		Value	Std Err	K_2_	q_ex_	q_the_
Na-B	0.9988	Slope	0.02425	0.0002214	0.005	40	41.23
		Intercept	0.1176	0.01461			
CPC-B	0.9827	Slope	0.03200	0.001136	0.0038	30.89	31.25
		Intercept	0.2676	0.08036			
Al-B	0.9814	Slope	0.05159	0.001833	0.0042	18.33	19.38
		Intercept	0.6250	0.1309			

Intraparticle diffusion model							
Clay	R^2^		Value	Std Err	K_id_	C	
Na-B	0.7843	Slope	2.738	0.3589	2.738	14.39	
		Intercept	14.39	2.565			
CPC-B	0.7688	Slope	1.835	0.2598	1.835	11.43	
		Intercept	11.43	1.933			
Al-B	0.8936	Slope	1.279	0.1103	1.279	5.018	
		Intercept	5.018	0.8310			

The correlation coefficients R^2^ for the pseudo-second-order kinetic model were found to be 0.99, 0.982 and 0,981 for Na-B, CPC-B and Al-B respectively indicating a strong agreement between the experimental values of q_exp_ and the calculated values q_the_, as presented in Table 2. This alignment suggests that the pseudo-second-order model is the most appropriate for characterizing the adsorption of rosemary essential oil onto the purified and the modified clays. Comparable findings were reported in studies examining the adsorption of terpenic compounds from Eucalyptus globulus essential oil onto beidellite, modified beidellite (Ghrab et al. 2018a), and organo-palygorskite (Ghrab et al. 2018b). These results imply that the adsorption mechanism is predominantly governed by surface control through chemisorption (Ghrab et al. 2018b).

### Determination of antioxidant activity

**Fig. 10:**
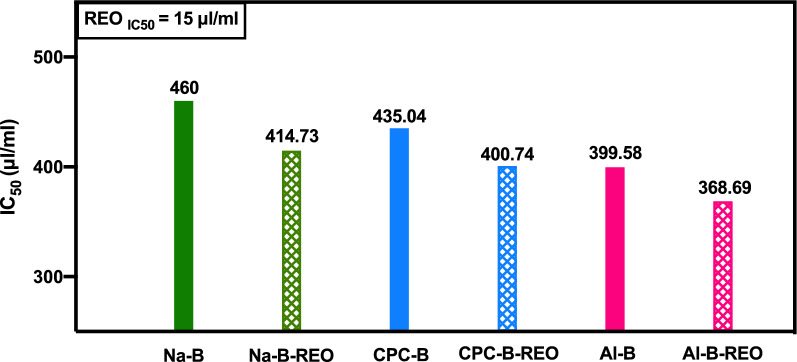
Antioxidant activity of encapsulated REO with Na-B, CPC-B and Al-B

Antioxidants are substances that retard the oxidation process by inhibiting the polymerization chain initiated by free radicals. The antioxidant activity of the adsorption and encapsulation media used in this study was carried out after adsorption/addition of rosemary essential oil, and the results are shown in Fig. 10.

With regard to formulation type, we observed that carriers containing bridged clay (Al-B) showed the highest antioxidant capacities 368.69 μl/ml, which may be attributed to the nature of the formulation. In this study, pillared clay showed the less value for both adsorption and desorption while the antioxidant activity for Al-B is higher than those of Na and CPC-B. This can be explained by the involvement of the Al Keggin ion which has been demonstrated to possess antioxidant properties in prior research (Lei et al. 2023), and the quality of the entrapped REO phenolic components responsible for the quenching of free radicals (Dimitrios 2006).

Clays are recognized not only for their role as carriers of antioxidants but also for exhibiting intrinsic antioxidant properties, which are influenced by their functionalization and modifications through the adsorption of oxidative species or redox reactions involving naturally occurring metals such as iron and manganese. Research conducted by Gutiérrez et al. (2017) indicates that montmorillonite clay demonstrates minimal antioxidant activity unless it is combined with active compounds. Conversely, Mylarappa et al. (2023) reported significant scavenging activity in clay when it is not associated with Ruthenium oxide nanocomposites, while Gul et al. (2023) observed that kaolin clay can also display antioxidant activity independently. Organically modified clays, particularly those treated with surfactants, enhance radical scavenging capabilities, especially for hydrophobic antioxidants, due to the presence of amino groups that significantly contribute to antioxidant efficacy (Omidi and Kakanejadifard 2019). Lastly, pillared clays enhance the antioxidant activity by facilitating electron transfer through redox reactions, which effectively reduces the IC_50_ value.

## Conclusions

The aim of studying the gas-phase adsorption of the active compounds used in this study was to compare the adsorption capacity of different clay supports in a state other than liquid. Adsorption in a liquid medium increases the contact surface as colloidal particles are suspended in the solution, allowing the solute to reach the inner surface and intercalate into the clay structure. This is probably due to the fact that the CPC surfactant occupies the available adsorption sites, reducing the adsorption area. Infrared analysis has clearly demonstrated the presence of phenolic groups, the imprint of active compounds, on clay materials.

This research revealed that essential oil molecules with hydrophilic functional groups were absorbed effectively, suggesting that the hydrophilic nature of the layered silicates played a crucial role in attracting these molecules. Consequently, layered silicates with increased hydrophilicity are considered to be the optimal choice for adsorbing essential oils in gaseous phase. The study also demonstrated that the size of the molecules and the presence of hydrophilic functional groups greatly influenced the gaseous adsorption levels of rosemary essential oil.

The novel adsorption method utilized in this research is devoid of chemical agents, illustrating the feasibility of a volatile compound being adsorbed onto a substrate. Importantly, this approach enables the establishment of robust bonds between the two components. As a result of this work, the Na-B clay/REO system obtained is a low cost, non-toxic and can be used in pharmaceutical, food and cosmetic industry.

## Data Availability

All data generated or analyzed during this study are included in this article.

## References

[CR1] Ali B, Al-Wabel NA, Shams S, Ahamad A, Khan SA, Anwar F (2015) Essential oils used in aromatherapy: a systemic review. Asian Pac J Trop Biomed 5:601–611. 10.1016/j.apjtb.2015.05.007

[CR2] Arif ZU, Khalid MY, Ahmed W, Arshad H (2022) A review on four-dimensional (4D) bioprinting in pursuit of advanced tissue engineering applications. Bioprinting 27:e00203. 10.1016/j.bprint.2022.e00203

[CR3] Avena MJ, Cabrol R, De Pauli CP (1990) Study of some physicochemical properties of pillared montmorillonites; acid-base potentiometric titrations and electrophoretic measurements. Clays Clay Minerals 38:356–362

[CR4] Bakkali F, Averbeck S, Averbeck D, Idaomar M (2008) Biological effects of essential oils–a review. Food Chem Toxicol 46:446–475. 10.1016/j.fct.2007.09.106 17996351 10.1016/j.fct.2007.09.106

[CR5] Baniasadi H, Fathi Z, Cruz CD, Abidnejad R, Tammela P, Niskanen J, Lizundia E (2025) Structure-property correlations and environmental impact assessment of sustainable antibacterial food packaging films reinforced with fungal chitin nanofibrils. Food Hydrocolloids 162:110987. 10.1016/j.foodhyd.2024.110987

[CR6] Berraaouan D, Essifi K, Addi M, Hano C, Fauconnier M-L, Tahani A (2023) Hybrid microcapsules for encapsulation and controlled release of rosemary essential oil. Polymers 15:823. 10.3390/polym15040823 36850108 10.3390/polym15040823 PMC9968220

[CR7] de Oliveira LH, Trigueiro P, Souza JSN, de Carvalho MS, Osajima JA, da Silva-Filho EC, Fonseca MG (2022) Montmorillonite with essential oils as antimicrobial agents, packaging, repellents, and insecticides: an overview. Colloids Surf B Biointerfaces 209:112186. 10.1016/j.colsurfb.2021.112186 34740094 10.1016/j.colsurfb.2021.112186

[CR8] Dimitrios B (2006) Sources of natural phenolic antioxidants. Trends Food Sci Technol 17:505–512. 10.1016/j.tifs.2006.04.004

[CR9] Elmiz M, Essifi K, Berraaouan D, Salhi S, Tahani A (2019) Adsorption thermodynamics and isosteric heat of adsorption of Thymol onto sodic, pillared and organic bentonite. Mediterr J Chem 8:494–504. 10.13171/mjc8619080210em

[CR10] Essifi K, Hammani A, Berraaouan D, El Bachiri A, Fauconnier M-L, Tahani A (2022) Montmorillonite nanoclay based formulation for controlled and selective release of volatile essential oil compounds. Mater Chem Phys. 10.1016/j.matchemphys.2021.125569

[CR11] França DB, Oliveira LS, Filho FGN, Filho ECS, Osajima JA, Jaber M, Fonseca MG (2022) The versatility of montmorillonite in water remediation using adsorption: current studies and challenges in drug removal. J Environ Chem Eng 10:107341. 10.1016/j.jece.2022.107341

[CR12] Ghrab S, Balme S, Cretin M, Bouaziz S, Benzina M (2018a) Adsorption of terpenes from *Eucalyptus globulus* onto modified beidellite. Appl Clay Sci 156:169–177. 10.1016/j.clay.2018.02.002

[CR13] Ghrab S, Eloussaief M, Lambert S, Bouaziz S, Benzina M (2018b) Adsorption of terpenic compounds onto organo-palygorskite. Environ Sci Pollut Res Int 25:18251–18262. 10.1007/s11356-017-9122-2 28500552 10.1007/s11356-017-9122-2

[CR14] Giannakas A, Tsagkalias I, Achilias DS, Ladavos A (2017) A novel method for the preparation of inorganic and organo-modified montmorillonite essential oil hybrids. Appl Clay Sci 146:362–370. 10.1016/j.clay.2017.06.018

[CR15] Gianotti V, Benzi M, Croce G, Frascarolo P, Gosetti F, Mazzucco E, Bottaro M, Gennaro MC (2008) The use of clays to sequestrate organic pollutants. Leaching Exp Chemosphere 73:1731–1736. 10.1016/j.chemosphere.2008.09.007 10.1016/j.chemosphere.2008.09.007 18929393

[CR16] Gul T, Khan I, Ahmad B, Ahmad S, Alsaiari AA, Almehmadi M, Abdulaziz O, Alsharif A, Khan I, Saeed K (2023) Efficient photodegradation of methyl red dye by kaolin clay supported zinc oxide nanoparticles with their antibacterial and antioxidant activities. Heliyon 9:e16738. 10.1016/j.heliyon.2023.e16738 37313164 10.1016/j.heliyon.2023.e16738 PMC10258418

[CR17] Gutiérrez TJ, Ponce AG, Alvarez VA (2017) Nano-clays from natural and modified montmorillonite with and without added blueberry extract for active and intelligent food nanopackaging materials. Mater Chem Phys 194:283–292. 10.1016/j.matchemphys.2017.03.052

[CR18] Haghighi H, Gullo M, La China S, Pfeifer F, Siesler HW, Licciardello F, Pulvirenti A (2021) Characterization of bio-nanocomposite films based on gelatin/polyvinyl alcohol blend reinforced with bacterial cellulose nanowhiskers for food packaging applications. Food Hydrocolloids 113:106454. 10.1016/j.foodhyd.2020.106454

[CR19] Hamed M, Monteiro CE, Sayed AEH (2022) Investigation of the impact caused by different sizes of polyethylene plastics (nano, micro, and macro) in common carp juveniles, *Cyprinus carpio* L., using multi-biomarkers. Sci Total Environ 803:149921. 10.1016/j.scitotenv.2021.149921 34482135 10.1016/j.scitotenv.2021.149921

[CR20] Ho YS, Wase DAJ, Forster CF (1996) Kinetic Studies of competitive heavy metal adsorption by Sphagnum Moss Peat. Environ Technol 17:71–77. 10.1080/09593331708616362

[CR21] Kadar F, Szazdi L, Fekete E, Pukanszky B (2006) Surface characteristics of layered silicates: influence on the properties of clay/polymer nanocomposites. Langmuir 22:7848–7854. 10.1021/la060144c 16922573 10.1021/la060144c

[CR22] Keawchaoon L, Yoksan R (2011) Preparation, characterization and in vitro release study of carvacrol-loaded chitosan nanoparticles. Colloids Surf B Biointerfaces 84:163–171. 10.1016/j.colsurfb.2010.12.031 21296562 10.1016/j.colsurfb.2010.12.031

[CR23] Kennedy KK, Maseka KJ, Mbulo M (2018) Selected adsorbents for removal of contaminants from wastewater: towards engineering clay minerals. Open J Appl Sci 08:355–369. 10.4236/ojapps.2018.88027

[CR24] Khalid MY, Arif ZU (2022) Novel biopolymer-based sustainable composites for food packaging applications: a narrative review. Food Packag Shelf Life 33:100892. 10.1016/j.fpsl.2022.100892

[CR25] Kim J-H, Shin WS, Song D-I, Choi SJ (2005) Multi-step competitive sorption and desorption of chlorophenols in surfactant modified montmorillonite. Water Air Soil Pollut 166:367–380. 10.1007/s11270-005-6329-5

[CR26] Kinninmonth MA, Liauw CM, Verran J, Taylor R, Edwards-Jones V, Shaw D, Webb M (2013) Investigation into the suitability of layered silicates as adsorption media for essential oils using FTIR and GC–MS. Appl Clay Sci 83–84:415–425. 10.1016/j.clay.2013.07.009

[CR27] Lagergren S (1898) About the theory of so called adsorption of soluble substances. Kungliga Svenska Vetenskapsakademiens Handlingar 24:1–39

[CR28] Lei S, Yang H, Li J, Li Y, Wang L, Chen B, Li J (2023) A study of the antioxidant properties of Keggin-type polyoxometalates. Dalton Trans 52:9673–9683. 10.1039/d3dt01361h 37382407 10.1039/d3dt01361h

[CR29] Ling Q, Zhang B, Wang Y, Xiao Z, Hou J, Xiao C, Liu Y, Jin Z (2022) Chemical composition and antioxidant activity of the essential oils of Citral-Rich Chemotype *Cinnamomum camphora* and *Cinnamomum bodinieri*. Molecules 27:7356. 10.3390/molecules27217356 36364183 10.3390/molecules27217356 PMC9656011

[CR30] MacEwan DMC (1948) Complexes of clays with organic compounds. I. Complex formation between montmorillonite and halloysite and certain organic liquids. Trans Faraday Soc 44:349–367. 10.1039/tf9484400349

[CR31] Majeed H, Bian Y-Y, Ali B, Jamil A, Majeed U, Khan QF, Iqbal KJ, Shoemaker CF, Fang Z (2015) Essential oil encapsulations: uses, procedures, and trends. RSC Adv 5:58449–58463. 10.1039/c5ra06556a

[CR32] Maji TK, Hussain MR (2008) Microencapsulation of Zanthoxylum limonella oil (ZLO) in genipin crosslinked chitosan–gelatin complex for mosquito repellent application. J Appl Polym Sci 111:779–785. 10.1002/app.29001

[CR33] Maurizzi E, Bigi F, Quartieri A, De Leo R, Volpelli LA, Pulvirenti A (2022) The green era of food packaging: general considerations and new trends. Polymers (Basel) 14(20):4257. 10.3390/polym14204257 36297835 10.3390/polym14204257 PMC9610407

[CR34] Moavenian MH, Yasrobi SS (2008) Volume change behavior of compacted clay due to organic liquids as permeant. Appl Clay Sci 39:60–71. 10.1016/j.clay.2007.04.009

[CR35] Mohamed EA, Hossein, Berraaouan D, Salhi S, Abdesselam T (2017) Chemical and physical characterization of Moroccan Bentonite taken from Nador (North of Morocco). Am J Chem 7:105–112. 10.5923/j.chemistry.20170704.01

[CR36] Mulla DJ (1985) Measurement of the specific surface area of clays by internal reflectance spectroscopy. Clays Clay Miner 33:391–396. 10.1346/ccmn.1985.0330503

[CR37] Mylarappa M, Chandruvasan S, Thippeswamy B, Shravana Kumara KN, Kantharaju S (2023) Clay incorporated ruthenium oxide nanocomposite for electrochemical, sensor, optical, photocatalytic and antioxidant studies. Sustain Chem Environ 2:100007. 10.1016/j.scenv.2023.100007

[CR38] Nguemtchouin MGM, Ngassoum MB, Chalier P, Kamga R, Ngamo LST, Cretin M (2013) *Ocimum gratissimum* essential oil and modified montmorillonite clay, a means of controlling insect pests in stored products. J Stored Prod Res 52:57–62. 10.1016/j.jspr.2012.09.006

[CR39] Nguemtchouin MGM, Ngassoum MB, Kamga R, Deabate S, Lagerge S, Gastaldi E, Chalier P, Cretin M (2015) Characterization of inorganic and organic clay modified materials: an approach for adsorption of an insecticidal terpenic compound. Appl Clay Sci 104:110–118. 10.1016/j.clay.2014.11.016

[CR40] Omidi S, Kakanejadifard A (2019) Modification of chitosan and chitosan nanoparticle by long chain pyridinium compounds: synthesis, characterization, antibacterial, and antioxidant activities. Carbohydr Polym 208:477–485. 10.1016/j.carbpol.2018.12.097 30658826 10.1016/j.carbpol.2018.12.097

[CR41] Parolo ME, Avena MJ, Pettinari GR, Baschini MT (2012) Influence of Ca2+ on tetracycline adsorption on montmorillonite. J Colloid Interface Sci 368:420–426. 10.1016/j.jcis.2011.10.079 22189389 10.1016/j.jcis.2011.10.079

[CR42] Parris N, Cooke PH, Hicks KB (2005) Encapsulation of essential oils in zein nanospherical particles. J Agric Food Chem 53:4788–4792. 10.1021/jf040492p 15941317 10.1021/jf040492p

[CR43] Paula HCB, Sombra FM, Cavalcante RdF, Abreu FOMS, de Paula RCM (2011) Preparation and characterization of chitosan/cashew gum beads loaded with Lippia sides essential oil. Mater Sci Eng 31:173–178. 10.1016/j.msec.2010.08.013

[CR44] Punia Bangar S, Ilyas RA, Chowdhury A, Navaf M, Sunooj KV, Siroha AK (2023) Bentonite clay as a nanofiller for food packaging applications. Trends Food Sci Technol 142:104242. 10.1016/j.tifs.2023.104242

[CR45] Saucedo-Zuñiga JN, Sánchez-Valdes S, Ramírez-Vargas E, Guillen L, Ramos-deValle LF, Graciano-Verdugo A, Uribe-Calderón JA, Valera-Zaragoza M, Lozano-Ramírez T, Rodríguez-González JA et al (2021) Controlled release of essential oils using laminar nanoclay and porous halloysite / essential oil composites in a multilayer film reservoir. Micropor Mesopor Mater 316:110882. 10.1016/j.micromeso.2021.110882

[CR46] Scalvenzi L, Durofil A, Cáceres Claros C, Pérez Martínez A, Guardado Yordi E, Manfredini S, Baldini E, Vertuani S, Radice M (2024) Unleashing nature’s pesticide: a systematic review of Schinus molle essential oil’s biopesticidal potential. Sustainability 16(23):10444. 10.3390/su162310444

[CR47] Shaaban HA, Farouk A. Encapsulation of essential oils and their use in food applications. In: Essential oils - advances in extractions and biological applications; Biochemistry; 2022. 10.5772/intechopen.103147.

[CR48] Solis M, Silveira S (2020) Technologies for chemical recycling of household plastics - a technical review and TRL assessment. Waste Manag 105:128–138. 10.1016/j.wasman.2020.01.038 32058902 10.1016/j.wasman.2020.01.038

[CR49] Srinivasan KR (1990) Use of inorgano-organo-clays in the removal of priority pollutants from industrial wastewaters: structural aspects. Clays Clay Miner 38:277–286. 10.1346/ccmn.1990.0380306

[CR50] Unalan I, Fuggerer T, Slavik B, Buettner A, Boccaccini AR (2021) Antibacterial and antioxidant activity of cinnamon essential oil-laden 45S5 bioactive glass/soy protein composite scaffolds for the treatment of bone infections and oxidative stress. Mater Sci Eng C Mater Biol Appl 128:112320. 10.1016/j.msec.2021.112320 34474871 10.1016/j.msec.2021.112320

[CR51] Weber WJ, Morris JC (1963) Kinetics of adsorption on carbon from solution. J Sanit Eng Div 89:31–59. 10.1061/jsedai.0000430

[CR52] Yalçın T, Alemdar A, Ece ÖI, Güngör N (2002) The viscosity and zeta potential of bentonite dispersions in presence of anionic surfactants. Mater Lett 57:420–424. 10.1016/s0167-577x(02)00803-0

